# Microwave-assisted biodiesel production using –SO_3_H functionalized heterogeneous catalyst derived from a lignin-rich biomass

**DOI:** 10.1038/s41598-023-36380-1

**Published:** 2023-06-05

**Authors:** Nidhi Yadav, Gaurav Yadav, Md. Ahmaruzzaman

**Affiliations:** Department of Chemistry, National Institute of Technology Silchar, Silchar, Assam 788010 India

**Keywords:** Renewable energy, Bioinspired materials

## Abstract

The synthesis of biodiesel from renewable resources has immense potential as a sustainable and cost-effective energy alternative. In this work, a reusable –SO_3_H functionalized heterogeneous catalyst that has a total acid density of 2.06 mmol/g was prepared from walnut (*Juglans regia*) shell powder by low-temperature hydrothermal carbonization (WNS-SO_3_H). Walnut shell (WNS) contains more lignin (50.3%), which shows great resistance toward moisture. The prepared catalyst was employed for the effective conversion of oleic acid to methyl oleate by a microwave-assisted esterification reaction. The EDS analysis revealed the significant presence of sulfur (4.76 wt%), oxygen (51.24 wt%), and carbon (44 wt%) content. The results of the XPS analysis confirm the bonding of C–S, C–C, C=C, C–O, and C=O. Meanwhile, the presence of –SO_3_H (the responsible factor for the esterification of oleic acid) was confirmed by FTIR analysis. Under the optimized conditions (9 wt% catalyst loading, 1:16 oleic acid to methanol molar ratio, 60 min reaction time, and 85 °C temperature), the conversion of oleic acid to biodiesel was found to be 99.01 ± 0.3%. The obtained methyl oleate was characterized by employing ^13^C and ^1^H nuclear magnetic spectroscopy. The conversion yield and chemical composition of methyl oleate were confirmed by gas chromatography analysis. In conclusion, it can be a sustainable catalyst because the catalyst preparation controls the agro-waste, a great conversion is achieved due to the high lignin content, and the catalyst was reusable for five effective reaction cycles.

## Introduction

The worldwide energy crisis is caused by the steady depletion of fossil fuel supplies, exacerbated by the increasing population in growing countries^1^. When the estimated demand for energy is compared to available crude oil supplies, it is clear that foreseeable demand for energy cannot be fulfilled entirely by fossil fuels. Furthermore, fossil fuels are not environmentally friendly since they are related to high toxicity, global warming, and non-biodegradability, making them a non-sustainable energy source^2^. Techniques for energy production, fuels, and feedstock materials using more sustainable methods have been developed due to growing consciousness about global climate change and the rising consumption of resources of fossil fuels^2^. As a result, it is unavoidable to fix the reliance on crude oil as well as the rising environmental degradation by building a competitive and sustainable substitute based on abundant and renewable feedstock such as biomass or other regenerative sources^1^. Numerous renewable energy sources may assist in reducing the growing ultimatum for energy. Biofuel is an alternative energy source commonly utilized to fulfill energy requirements. It is, in particular, a long-term option for reducing fossil fuel dependence and environmental concerns^3^. However, existing renewable energy technology for biofuel production is highly expensive than conventional fossil fuels. This may be due to the fact that the technology is novel and expensive to implement. In contrast, biomass is readily accessible worldwide for biofuel manufacturing, such as biodiesel, and it is less expensive than other renewable energy sources^4^. Therefore, a low-cost solution is biomass to replace conventional fuels with transesterification and esterification of free fatty acids for biodiesel synthesis^5^.

Recently, rapid growth has been seen in biodiesel production and its utilization, because it has the ability to bring down the outpouring of greenhouse gases (GHG)^6^. Biodiesel is a sulfur-free alternative to diesel that offers benefits like biocompatibility, degradability, innocuous nature, safe operation, ease of synthesis, less emission of GHG, high flash point, higher cetane number, and intrinsic lubrication when compared to oil-derived diesel^7,8^. Generally, biodiesel is made by trans-esterifying oils with short-alkyl chain alcohols like methanol or ethanol in the presence of an appropriate catalyst^9^. When biodiesel production is performed using vegetable oils, pre-processing is necessary before transesterification because oils contain very high free fatty acids and moisture levels^10^. Thus, it could result in soap production during the transesterification process of oils with base catalysts. The conversion of FFAs to alkyl esters by an acid-catalyzed esterification process lowers the FFA concentration^11^.

However, homogeneous acidic and basic catalysts such as NaOH, KOH, H_2_SO_4_, and H_3_PO_4_ are broadly employed to synthesize biodiesel due to their great catalytic performance and lower reaction temperature^12–14^. The usual manufacturing of biodiesel using homogenous acid catalysts has many major challenges, such as contamination of the biodiesel due to sulfur content, reactor corrosion, soap formation, and the formation of numerous pollutant molecules. Therefore, to overcome all the related issues, heterogeneous acid catalysts are grabbing substantial interest because of their non-corrosive and non-toxic nature, moisture resistance, reusability, and easy separation^15^.

Recently, SFHCs have gained high attention because of their high usability for different applications such as esterification^3^, transesterification^16^, solketal synthesis^17^, water decontamination^18^, and other organic transformations^19–22^. Agricultural waste-derived SFHCs have been exploited as effective green catalysts in biodiesel production without further chemical treatment^23^. SFHCs derived from biomass have numerous specific characteristics, such as providing excellent conversion efficiency, being easily separable from the reaction mixture, and being highly reusable, cost-effective, and environmentally friendly. Numerous SFHCs, including, Areca nut husk^24^, Acai stone^25^, citrus limetta peel^26^, Sugarcane bagasse^27^, and Sargassum horneri^28^, Pineapple leaves^29^, Date pits^30^, Tucuma peels^31^ have been reported for biodiesel production. However, these existing catalysts have several problems, and they provide lower ester content in biodiesel with harsh reaction conditions, which affect the cost of biodiesel production. All these catalysts are fabricated from cellulosic biomass, but lignin-rich biomass has yet to be effectively explored for catalyst fabrication. Lignin is the second carbon-rich material in nature after cellulose^32^.

Lignocellulosic material is a naturally occurring substance generated in considerable quantities each year. It was calculated that farmland plants produce (170–200) × 10^9^ tonnes of biomass annually, with around 70% of that coming from plant cell walls. Lignocellulose is easily accessible as a component of many waste streams from industries including agriculture, forestry, and the paper industry and does not conflict with the food business. As a result, lignocellulose is most likely to replace carbon as the primary renewable carbon source in the future^33^. Every year, approximately 89,000 tonnes of walnut kernels are produced, while approximately 2,000,000 tonnes of walnuts (in the shell) are produced^34^. As a consequence of the processing of walnuts, a considerable quantity of discarded walnut shells is produced, which may be a potential energy source. In the chemical composition of walnut shells, lignin is present in comparatively higher content, whereas hemicellulose and cellulose are present in lower content^35^. Therefore, as lignin is hydrophobic in nature, it shows resistance toward moisture, resulting in an “energy content” equivalent to coal^33,36^. Activated carbon made from lignocellulosic biomass typically has a large pore volume and surface area and is extensively utilized as a catalytic support and absorbent. The excellent permanence and high catalytic activity of lignin-derived catalysts or catalytic supports are attributable to their 3-D interpenetrating polymer network skeleton and aromatic units^14^. Therefore, an SFHC has been fabricated using walnut shell powder as a source of biomass.

In the industry of chemical synthesis, microwave heating is extensively used because of the potential to increase the rate of reaction and yield of a product, as well as because it is safe for heating reactants mixes at high temperatures. In comparison to the conventional heating method, the microwave heating method for chemical reactions is based on the transfer of energy via EMRs (electromagnetic radiation) because it requires less time as well as less energy supply for heating^24,37^. Microwave radiation is non-ionizing electromagnetic waves containing wavelengths ranging from 1 mm to 1 m and frequencies ranging from 0.3 to 300 GHz^38^. Heat production was detected during the reaction, mostly as a result of the high-frequency rotation of alcohol in constantly changing magnetic and electric fields, also known as dipole rotation. Furthermore, the oscillation of ions in the solution slows down and shifts direction in response to a fluctuating electric field, generating heat through conduction. Dielectric heating refers to these two phenomena^39^. Methanol has a polar nature and, having a high dielectric constant is favored for microwave-assisted esterification reactions^40^.

The performance of SFHC derived from walnut shells for biodiesel production has not been reported yet; however, there is a lot of potential for developing a catalyst for energy production using the walnut shell. In this study, a series of SFHCs were fabricated using the hydrothermal carbonization method at low temperatures. The main objective of the present work is to perform a microwave-assisted esterification reaction of oleic acid with methanol to produce the highest yield of biodiesel. The effect of various factors affecting biodiesel production, such as reaction time, catalyst dose, temperature, alcohol, and acid ratio, was also investigated. It was observed that the WNS-SO_3_H catalyst showed a conversion efficiency of 99.01 ± 0.3%, which is much higher than the previously reported literature. Additionally, a comparative study between the conventional method and microwave-assisted esterification reaction for biodiesel production was also performed, which marks the novelty of this research. The reusability study shows that the prepared catalyst is highly stable and reusable up to five cycles suggesting that the WNS-SO_3_H could be a potential catalyst for biodiesel production.

## Materials and methods

### Materials

Walnuts were purchased by the local market in Silchar, India. After using the walnut kernels, leftover walnut shells were used for the catalyst preparation. Sigma Aldrich provided oleic acid (OA, 99%) for this study. Concentrated sulfuric acid (98.06%), barium chloride (99.95%), methanol (99.8%), phenolphthalein (98%), NaCl (98%), and sodium hydroxide (97.79%) were purchased from Merck. All chemicals were used as they were obtained without additional purification. To get distilled water, the Simplicity® UV Water Purification System (Merck) has been used.

### Preparation of catalyst

**Figure 1:**
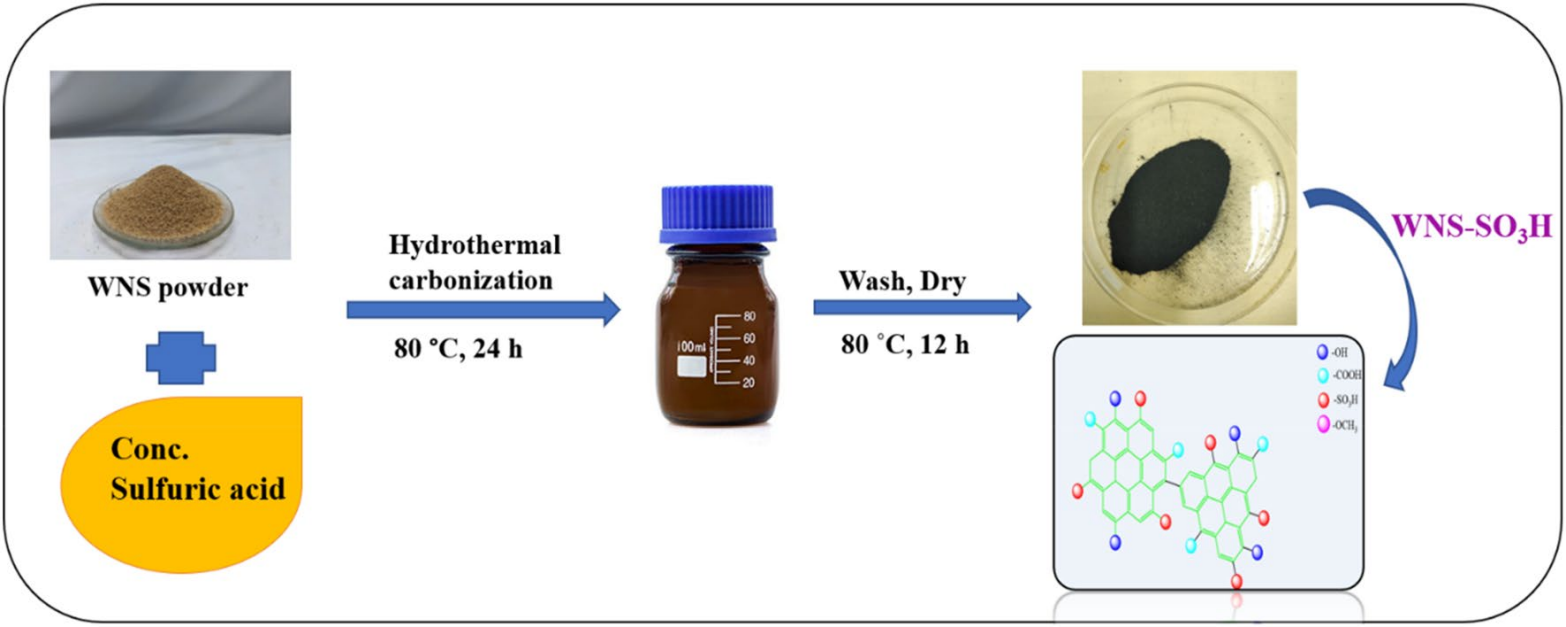
Preparation of WNS-SO_3_H by hydrothermal carbonization method.

Walnut shells were rinsed with distilled water and dried in a hot air oven for 30 h at a temperature of 80 °C. Oven-dried walnut shells are then grounded into powder using an electric grinder. The walnut shell powder was sieved through a 125-micron stainless steel sieve. After that, the measured amount of walnut powder was taken in four different borosilicate bottles (2 g in each bottle). Different proportions of concentrated sulphuric acid 1:10, 1:13, 1:17, and 1:20 (10.62, 13.82, 18.05, and 21.27 mL respectively) were added in the borosilicate bottles, and the resultant mixture was stirred for 30 min to make the homogeneous solution. After that, these bottles were kept in the oven at 80 °C for 24 h. The final mixture is cooled down and diluted with distilled water and washed multiple times to remove excess sulfate. The presence of sulfate ions was checked using a BaCl_2_ solution. After washing, the catalyst was dried in the oven at 80 °C for 12 h (Fig. 1).

The synthesized catalysts were stored in airtight vials and named WNS-1 (1:10), WNS-2 (1:13), WNS-3 (1:17), and WNS-4 (1:20). Further CHNS (Carbon, Hydrogen, Nitrogen, and Sulfur) elemental analyses were performed to determine the elemental composition of the prepared catalyst (Table 1). SO_3_H is the most important component for catalyst activity in the esterification reaction. Consequently, WNS-4 was taken to perform the esterification of oleic acid and renamed WNS-SO_3_H due to the high acid density.

**Table 1:** CHNS analysis of WNS batch catalysts along with their total acid density.

SI. no	Catalyst name	N%	C%	S%	H%	Total acid density (mmol/g)
1	WNS-1	0.41	42.03	1.90	0.49	1.31
2	WNS-2	0.59	45.54	2.46	0.75	1.58
3	WNS-3	0.41	40.75	3.07	2.48	1.92
4	WNS-SO_3_H	0.80	44.48	4.56	2.98	2.06

### CHNS analysis

The CHNS analysis of WNS-1, WNS-2, WNS-3, and WNS-SO_3_H catalysts was performed to determine the elemental composition. To perform the analysis 10 mg powder sample of each catalyst was taken in a tin capsule and placed in an autosampler. The tin boat enclosing the sample falls into the reactor chamber where excess oxygen was introduced before (at about 1150 °C). The complete oxidation is reached with a tungsten trioxide catalyst which is passed by the gaseous reaction products (CO_2_, H_2_O, NO_2,_ NO_3_. and SO_2_). The product gas mixture flows through a silica tube packed with copper granules. In this zone at 850 °C excess oxygen is bound and nitric/nitrous oxides are reduced. The leaving gas stream includes CO_2_, H_2_O, N_2_, and SO_2_, and was adsorbed at appropriate traps. High-purity helium was used as carrier gas. Finally, the gas mixture was passed to a gas chromatographic system. Separation of the elemental species (carbon, hydrogen, nitrogen, and sulfur) was done and detected using Thermal Conductivity Detector.

### Determination of acid density

Acid–base back titration was performed to determine the total acid density of the synthesized catalyst^24^. The following steps are used to calculate the total acid density of the catalysts; In a 50 mL conical flask, 0.05 g of each catalyst was mixed with 2 M aqueous solution of NaCl (15 mL). The resultant mixture is sonicated for 30-min using an ultrasonic sonicator to sonicate the suspension. After that, the solution was filtered, and the filtrated solution was titrated with an aqueous solution of 0.02 M NaOH solution using a phenolphthalein indicator^41,42^. Each titration result has been triplicated and their concordant value is noted. The formula for calculating the acid concentration is as follows:

1 $$C({H}^{+})=C{(OH}^{-})\frac{\Delta V}{m}$$

Here, OH^−^ is the NaOH concentration, m is the catalyst mass, and V is the titrant volume. The acid density of all four catalysts has been checked using the same procedure and it has been found that WNS-4 (1:20) has the highest acid density 2.06 mmol/g.

### Characterization of WNS-SO_3_H

Powder X-ray diffraction (XRD) spectroscopy, Fourier Transform Infra-Red (FTIR), Spectroscopy, scanning electron microscopy (SEM), X-ray energy dispersion spectroscopy (EDS), X-ray photoelectron spectroscopy (XPS), and CHNS analyzer, were used to characterize the WNS-SO_3_H catalyst. The ELEMENTAR vario EL III device was employed as a CHNS analyzer to determine the percentage amount of carbon, hydrogen, nitrogen, and sulfur. The XRD pattern in the catalyst was studied in a Philips X'pert Pro diffractometer equipped with Cu-K with a scanning rate of 2° min^−1^ and a range of 2θ between 10° and 90°. The functional groups in the catalyst were identified using FT-IR spectroscopy. For FT-IR spectroscopy in the 4000–400 cm^−1^ range, a Vertex 80 equipped with a Bruker 3000 Hyperion Microscope spectrometer and KBr pellets was used. For SEM–EDS; Jeol 639OLA/OXFORD XMX N was employed to look over the surface morphology of the WNS-SO_3_H. SEM–EDS was performed using an acceleration voltage of 0.5–60 eV and an EDAX detector area of 30 mm^2^. The XPS was performed on a Thermofisher Scientific Nexsa base model equipped with Auger electron microscopy, which has an ionization energy range of 21.2–40.8 eV. The TGA analysis was performed using TGA-DTA Hitachi STA7000 in the temperature range of 30–600 °C.

### Activity evaluation of WNS-SO_3_H

**Scheme 1:**
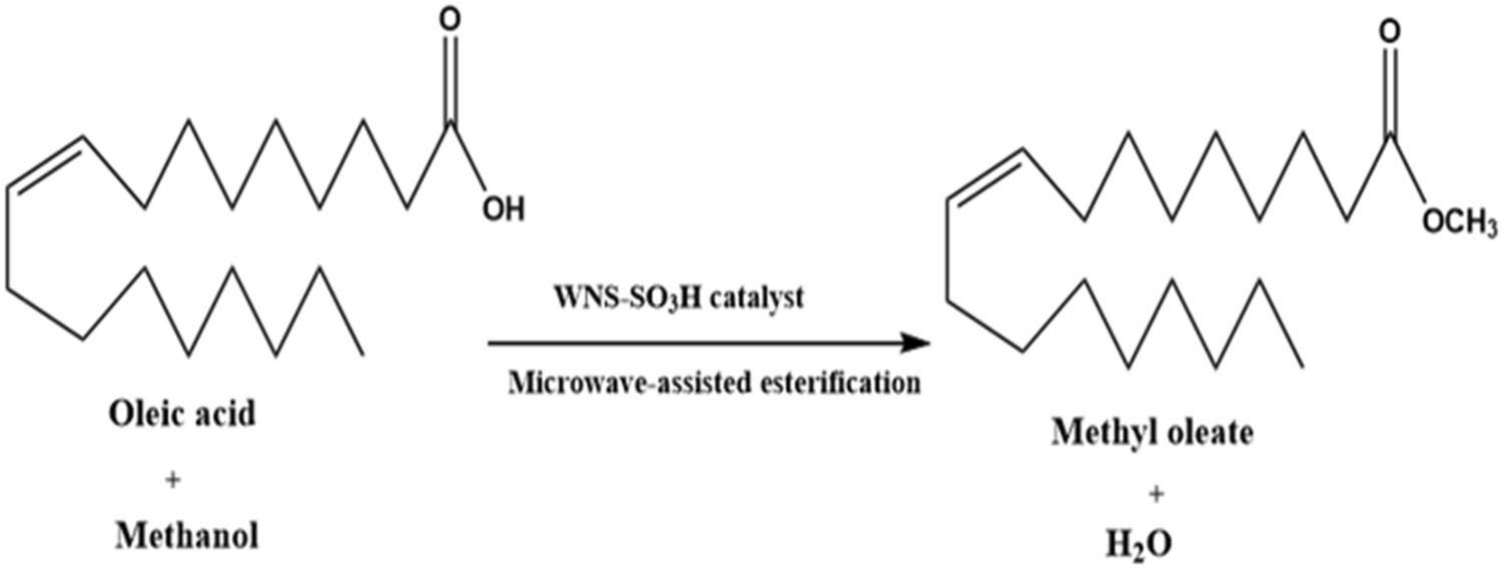
Microwave-assisted synthesis of methyl oleate (Biodiesel).

To evaluate the catalytic activity of WNS-SO_3_H, microwave-assisted esterification was performed. In a 15 mL ace pressure tube, WNS-SO_3_H catalyst (5–11 wt%) was taken in a homogenous mixture of oleic acid:methanol molar ratio (1:12–1:24) using 50 W microwave power at temperature (65–95 °C) using reaction time (60–100 min) to produce methyl oleate as a product (biodiesel) (Scheme 1). The optimum reaction conditions were catalyst dose 9 wt% (0.025 g), oleic acid to methanol molar ratio 1:16 (oleic acid 1 mmol (0.282 g):methanol 16 mmol (0.512 g), 85 °C temperature, and 60 min reaction time. Because the esterification reaction is an endothermic process, increasing the reaction temperature to an optimal value enhances biodiesel production by improving the rate of the reaction and shortening the reaction time^12^. The esterification reaction was carried out using a microwave (CEM, DISCOVER SYSTEM, DY 1073, 180/264 VAC.). Using the initial substrates and precoated silica gel (60 F254-Merck) plates, TLC (thin-layer chromatography) was employed to check the reaction's progress. After the reaction, the catalyst was isolated from the reaction mixture using filtration by Whatman paper, and methanol was employed to rinse the catalyst to remove impurities. A rotary evaporator was used to remove excess methanol from the reaction mixture and extract pure biodiesel.

### Characterization of biodiesel

After the extraction of pure biodiesel from the rotary evaporator under optimal conditions, a considerable amount of biodiesel is characterized using ^1^H and ^13^C nuclear magnetic resonance spectroscopy to confirm the formation of biodiesel (methyl oleate). ^1^H and ^13^C nuclear magnetic resonance spectroscopy was carried out in a Jeol instrument having model number ECZ500R/S1 having a frequency ranging from 10 to 535 MHz. The conversion of oleic acid into methyl oleate is obtained using Eq. (2).

2 $$Conversion \left(\%\right)= \frac{{2A}_{OMe}}{{3A}_{CH2}} \times 100$$

In this equation (Eq. 1), A_OMe_ stands for the integration of the OMe groups in FAME, and A_CH2_ stands for the total integration of the –CH_2_ (OA + FAME).

In order to verify the findings of the NMR investigation, a GC (gas chromatography) analysis was performed. GC was carried out using an Agilent model 8890 equipped with split/splitless injectors, polar columns (HP-5), and a UI Agilent column having diameter 30 m × 0.320 mm. The temperature of the oven was raised from 300 to 400 °C where the injection temperature was 300 °C. An external calibration curve has been prepared by methyl oleate (purchased from Merck) to quantify the peaks.

### Catalyst leaching and reusability

To look over the reusability of the WNS-SO_3_H catalyst, the esterification process was carried out by following the optimum conditions (9 wt% WNS-SO_3_H loadings, 1:16 oleic acid:methanol molar ratio, 85 °C reaction temperature, and a reaction period of 60 min) for the five effective cycles of reaction. After every cycle of esterification, the catalyst was separated from the mixture using filtration and rinsed using methanol for 4 times to eliminate the impurities from the surface of the catalyst. After that, the catalyst was oven dried at 80 °C for 4 h and used in further cycles.

Leaching test of WNS-SO_3_H catalyst performed by mixing catalyst with methanol solution. The solution is stirred using a magnetic stirrer for 6 h. After this, the solution was filtered and the resultant methanol solution is added with oleic acid under the optimum condition (85 °C reaction temperature, and a reaction period of 60 min).

### Kinetics of reaction

The chemical kinetics of WNS-SO_3_H catalyzed esterification of oleic acid with methanol can be explained using the following assumptions.

1. The rate of reaction of esterification of oleic acid was regulated by chemical reaction.
2. Since methanol is used in excess to carry out the reaction, its concentration may be taken to be constant.
3. The equilibrium of the reaction is shifted in the direction of FAME production because of the high concentration of methanol.

Therefore, the esterification is assumed to be a pseudo-first-order reaction^24,43^. The rate of the reaction between oleic acid and methanol can be shown by Eq. (3).

3 $$r=-\frac{d\left[Oleic\; acid\right]}{dt}=k\;[oleic \;acid]$$

where k = rate constant, t = reaction time, and [oleic acid] represents the oleic acid concentration. To determine the first-order rate constant by monitoring methyl ester conversion t was varied in Eq. (4).

4 $$-\mathrm{ln}\left(1-X\right)=kt$$

The activation energy of the esterification reaction was calculated at different temperatures: 55, 65, 75, and 85 °C. The Arrhenius Eq. (5) was used to calculate the energy of activation at different temperatures (55–85 °C)^44^.

5 $$lnk= -\frac{Ea}{RT}+lnA$$

## Results and discussion

### Powder X-ray diffraction analysis

**Figure 2:**
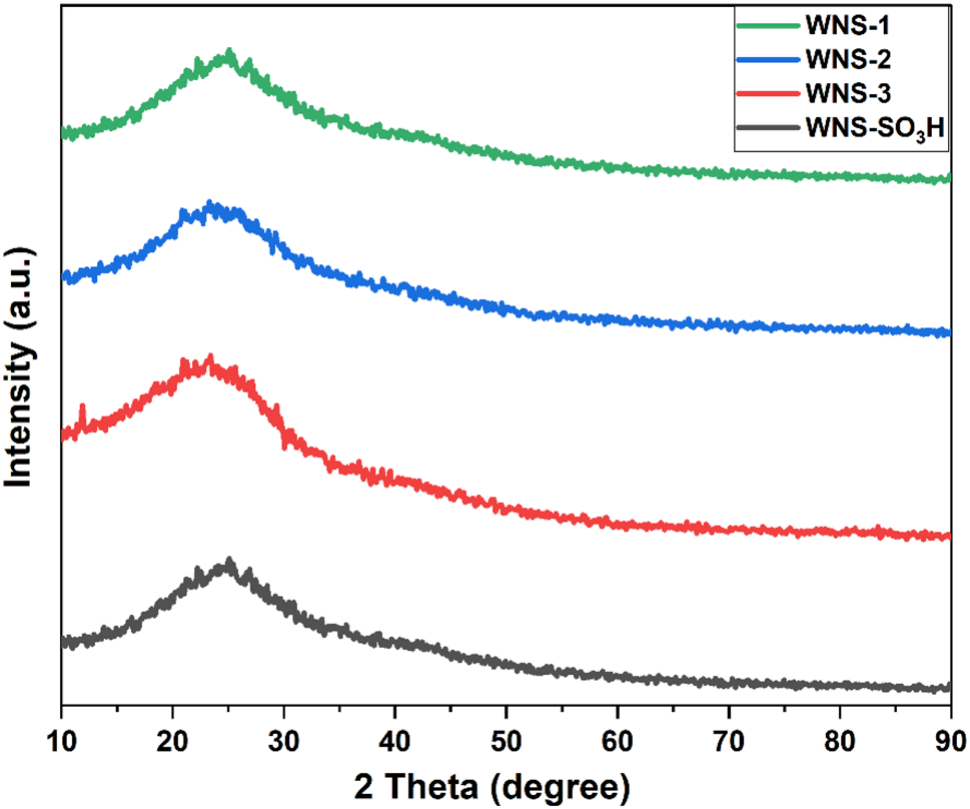
XRD pattern of WNS-1, WNS-2, WNS-3, and WNS-SO_3_H catalyst.

Figure 2 depicts the XRD pattern of all synthesized catalysts including the WNS-SO_3_H catalyst. Broad diffraction peaks of the catalysts suggest the amorphous nature. The large diffraction peaks obtained at 2θ = 20°–28° are responsible for the amorphous carbon structures that comprise the random orientation of aromatic carbon sheets^3,11^. A weak broad diffraction peak at 2θ = 43° represents the presence of a small amount of graphite carbon^12^. The analysis reveals that sulfonation (different molar ratios of sulfuric acid used for catalyst synthesis) does not affect the structure of the carbon skeleton, consequently, the carbonaceous structure of all prepared catalysts is the same.

### X-ray photoelectron spectroscopy

**Figure 3:**
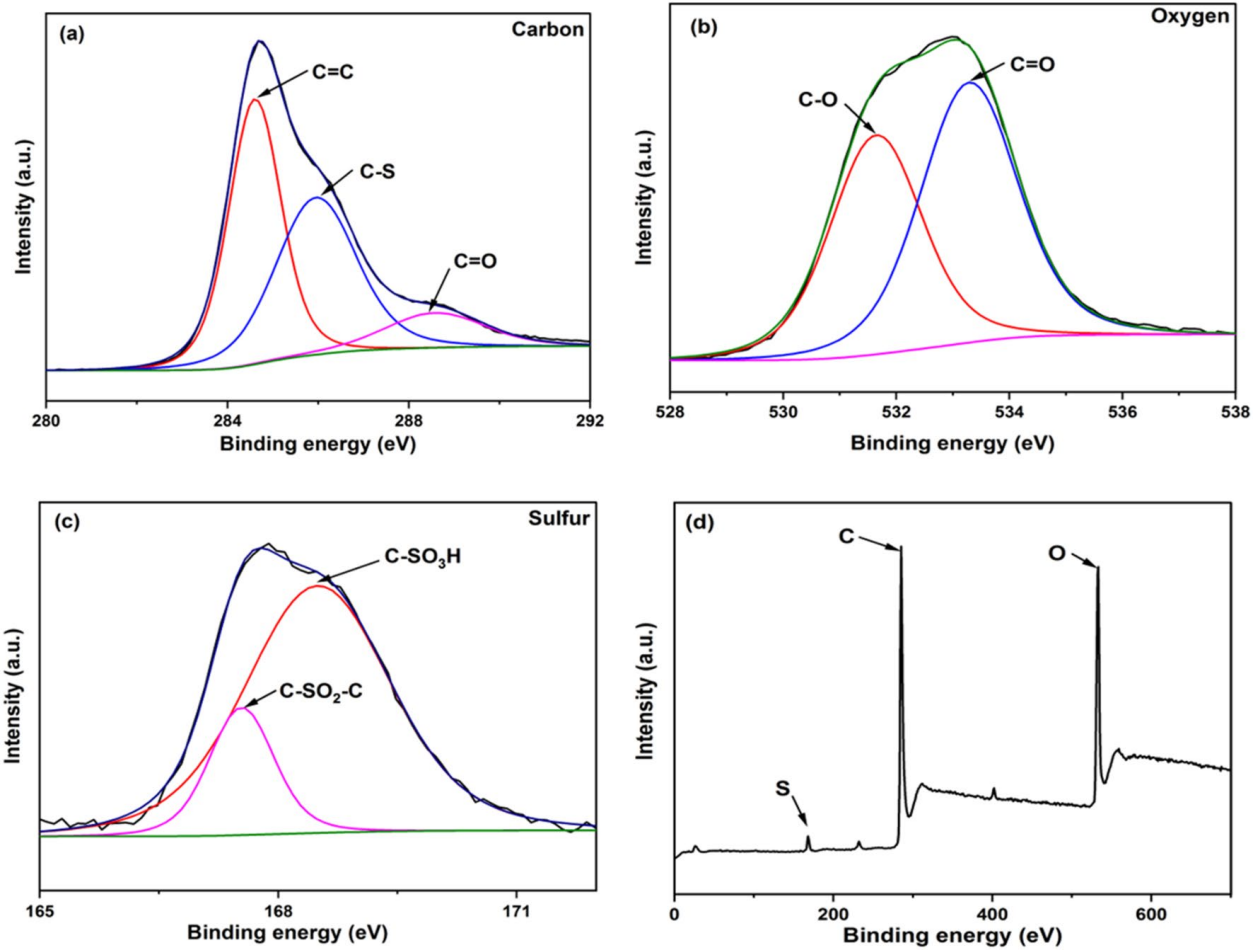
Deconvoluted XPS spectra of WNS-SO_3_H catalyst (**a**) Carbon spectrum (**b**) Oxygen spectrum (**c**) Sulfur spectrum (**d**) Overall XPS spectrum.

XPS analysis was performed to look into the chemical valences of the surface group on the surface of WNS-SO_3_H. Figure 3a shows the carbon spectrum, which consists of three peaks at 284.5, 286.7, and 288.9 eV, which is related to C=C, C–S, and C=O bonds respectively^25^. The oxygen spectrum shown in Fig. 3b was found to have two peaks at 531.9 eV related to C–O and 533.9 eV related to C=O. Figure 3c represents the sulfur spectra with two peaks at 167.3 and 168.8 eV, which represent the presence of C–SO_2_–C and C–SO_3_H bonding^25^. Furthermore, Fig. 3d displays the overall XPS spectrum of the catalyst WNS-SO_3_H. Therefore, the XPS analysis indicates that carbon, oxygen, and sulfur were present in the catalyst and also confirms that most of the sulfur is present as –SO_3_H, however, some content is also found in the form of SO_2_.

### SEM–EDS analysis

**Figure 4:**
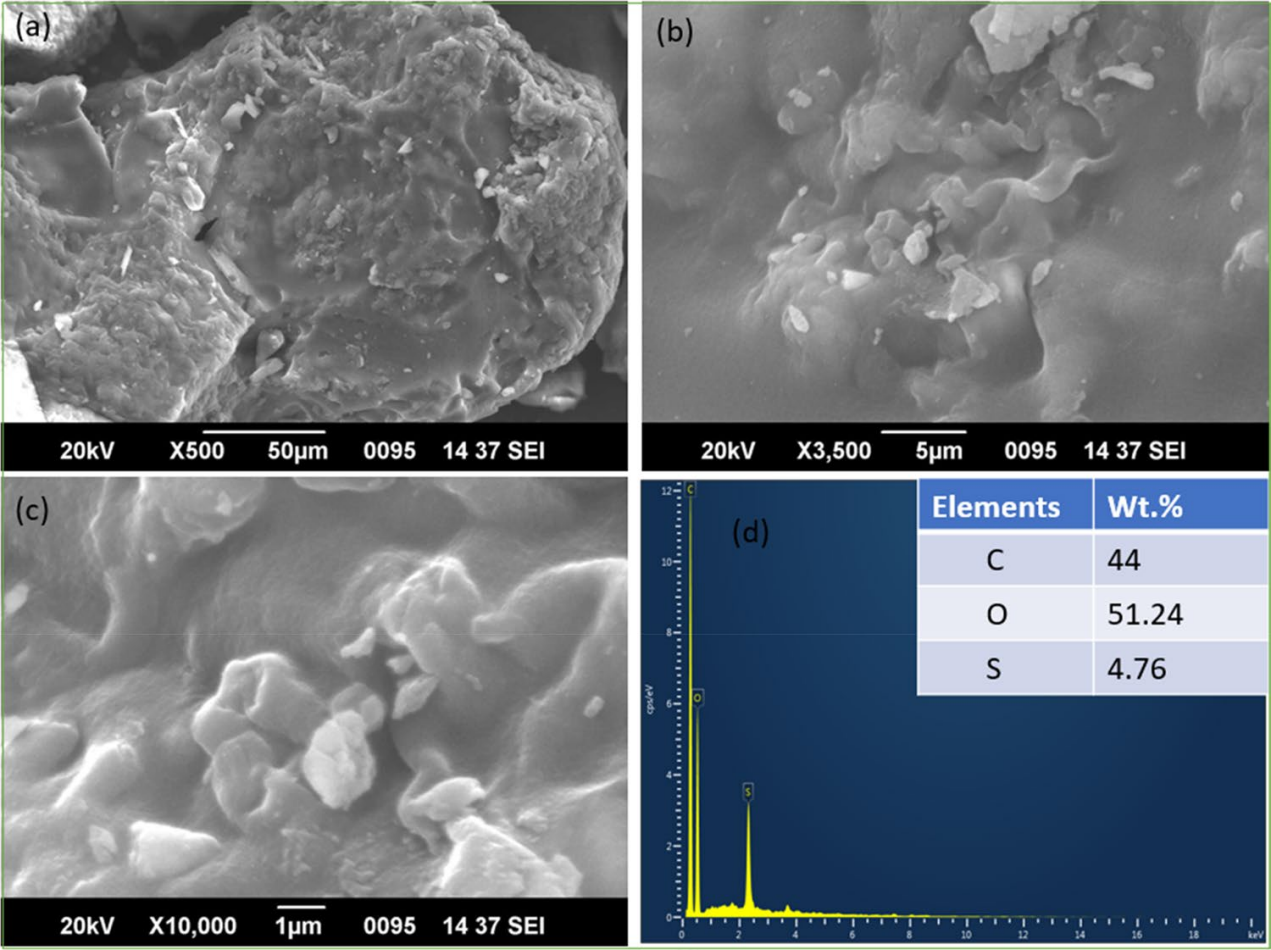
SEM images at different magnifications (**a**–**c**) of WNS-SO_3_H catalyst. (**d**) EDS analysis represents the elemental composition of the WNS-SO_3_H catalyst.

Scanning electron microscopy and X-ray energy dispersion spectroscopy were performed on WNS-SO_3_H to evaluate the structural morphology and composition of elements, respectively. SEM images in Fig. 4a–c are corresponding to the structural morphology of WNS-SO_3_H at different magnifications. SEM images reveal the non-pores and irregular morphology of the catalyst. The insertion of –SO_3_H groups block the surface pores resulting in the non-pores structure and showing the effective sulfonation of the catalytic surface. The surface morphology of the WNS-SO_3_H catalyst is similar to the previously reported studies that favor the effectiveness of the catalyst^45,46^. Additionally, EDS analysis was carried out to determine the elemental composition of the catalyst. Figure 4d reveals the composition of elements (Carbon 44 wt%, Oxygen 51.24 wt%, and Sulfur 4.76 wt%) present in WNS-SO_3_H. The significant presence of sulfur content reveals the catalytic activity of WNS-SO_3_H.

### Fourier transform infra-red spectroscopy

**Figure 5:**
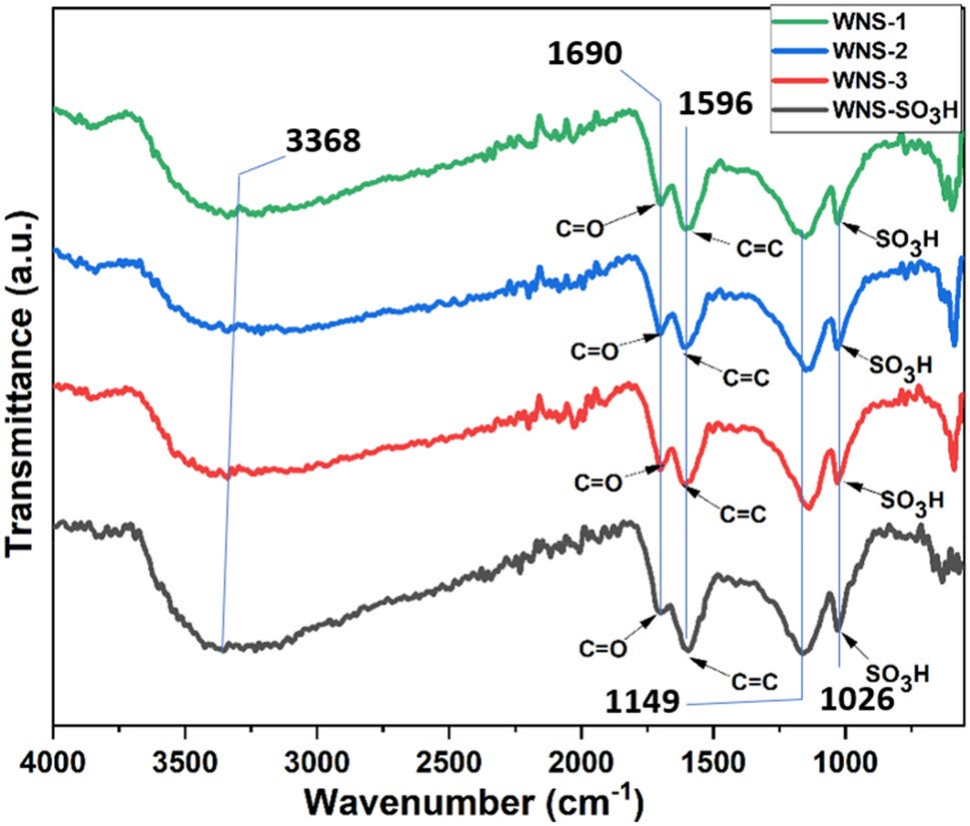
FTIR spectrum of WNS-1, WNS-2, WNS-3, and WNS-SO_3_H catalyst.

The presence of an –SO_3_H group at the surface of the WNS catalyst was confirmed by FTIR analysis portrayed in Fig. 5. The peaks at 1026 cm^−1^ and 1149 cm^−1^ correspond to the symmetric stretching of –SO_3_H groups and O=S=O bonding, respectively. The absorption band at 1596 cm^−1^ represents the C=C bonding of polyaromatic skeletal. The peak at 1690 cm^−1^ is related to the C=O bond from the –COOH group^25^. These findings corroborate the existence of acidic groups –COOH and –SO_3_H groups. The –SO_3_H groups substitute hydrogen on the solid surface to covalently attach to the carbon structure^47^. The peak at 3368 cm^−1^ is related to the –OH group existing at the catalyst’s surface.

### Thermogravimetric analysis

**Figure 6:**
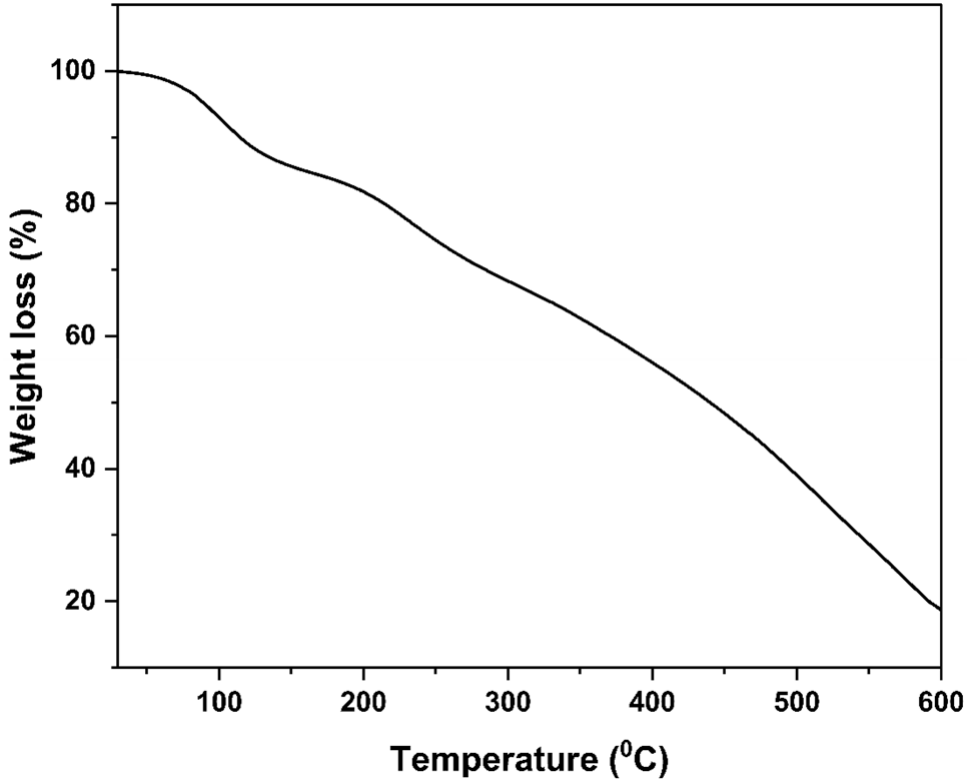
TGA pattern of WNS-SO_3_H catalyst.

The TGA analysis of the WNS-SO_3_H catalyst is shown in Fig. 6. The WNS-SO_3_H catalyst gives gradual weight loss with two decomposition stages at 150 and 250–300 °C. A significant weight loss was observed at 150 °C, which might be due to the removal of water content. The second decomposition stage of the catalyst occurred between 250 and 300 °C, which reveals the loss of –SO_3_H groups and cellulose degradation in the form of moisture (H_2_O) and gases (NH_3_ and CO_2_). After 300 °C weight loss in the WNS-SO_3_H catalyst was gradual, which was due to the lignin decomposition^48^.

### Factors affecting esterification reaction

#### Catalyst loading

**Figure 7:**
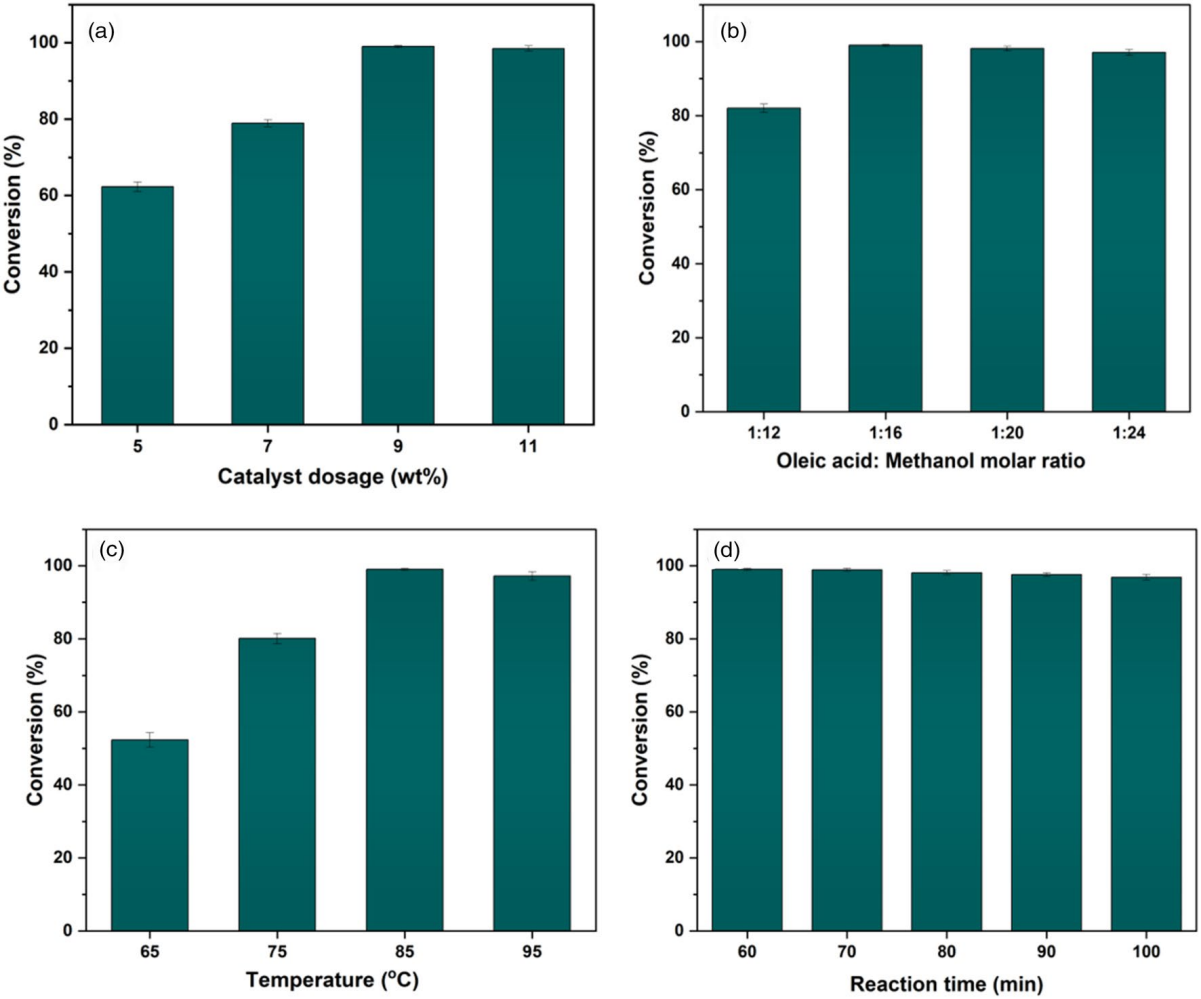
Factors affecting the esterification process. (**a**) Effect of catalyst loading. (**b**) Effect of oleic acid:methanol molar ratio. (**c**) Effect of reaction temperature. (**d**) Effect of reaction time.

The catalyst amount required to perform the maximum conversion affects the efficacy of the catalyst. To evaluate the effect of catalyst dosage, the catalyst concentration was regulated from 5 to 11 wt% in relation to the mass of oleic acid. It has been observed that when the catalyst concentration was raised from 5 to 9 wt%, there was a continuous increment in conversion, demonstrating that the quantity of catalyst had a substantial impact on the conversion of oleic acid. Because as the catalyst concentration increases, sulfur content also increases, which provides more reaction sites that result in high conversion. Nevertheless, when catalyst loading was increased by more than 9 wt% showed slightly reduced conversion (Fig. 7a). Since esterification is a reversible reaction, therefore an excess amount of catalyst may promote the reversible esterification reaction, consequently, overall product efficiency has been decreased^31,49^. Therefore, 9 wt% catalyst concentration has been taken as the optimum dose of the catalyst.

#### Oleic acid:methanol ratio

Esterification is a reversible reaction, and to initiate the reaction, an excess amount of methanol is frequently used to increase the solubility of the fatty phase with the phase of methanol. Furthermore, an excess of methanol may not result in improved catalytic efficiency since a considerable quantity of alcohol might limit oleic acid accessibility to the active sites of the catalyst. Therefore, a desired molar ratio of methanol is required for the esterification to carry out the reaction in a forward direction^27,30^. The impact of oleic acid to methanol molar ratio on conversion yield was investigated by changing the molar ratio to 1:12, 1:16, 1:20, and 1:24, at 85 °C for 60 min and 9 wt% catalyst doses. As the ratio of oleic acid to methanol went from 1:12 to 1:16, the conversion yield increases intensely. However, when the ratio went from 1:16 to 1:20, there was no significant change. which may be ascribed to reaction phase equilibrium. Furthermore, on increasing the ratio to 1:24, it has been observed that the conversion yield was decreased (Fig. 7b). Therefore, a 1:16 molar ratio was determined as the optimum oleic acid:methanol for the esterification reaction, and further reactions were performed with this ratio. It is worth mentioning that lower ratios, such as 1:16, may be used to achieve significant conversion without adversely affecting the activity of the catalyst. Because less alcohol is needed to drive the reaction and manufacturing costs would be also reduced^50^.

#### Reaction temperature

To look over the temperature effect on the esterification reaction, the temperature was changed from 65 to 95 °C. In the meantime, the remaining parameters were fixed at 9 wt% catalyst loading, a 1:16 oleic acid:methanol molar ratio, and 60 min of reaction time. A remarkable increment in conversion yield was noticed when the temperature was raised from 65 to 75 °C and reached equilibrium at 85 °C. By enabling mass transfer between the reactants and the surface of the catalyst, the molecules' increased kinetic energy causes the reaction to proceed more quickly as the temperature rises. The conversion was reduced by very high temperatures because they accelerate the methanol evaporation, which reduces the quantity of methanol that is present for the reaction with oleic acid^51^. As a result, conversion yield diminished with a further increase in temperature to 95 °C (Fig. 7c). Consequently, it was determined that the optimum temperature for the reaction was 85 °C. The reaction temperature for the esterification process is very less in comparison to similar studies, that used 100–150 °C reaction temperature^52,53^.

#### Reaction time

Time is a determining factor that greatly influences the catalyst's activity during biodiesel formation^52^. Reaction times were changed from 60 to 100 min to explore the effect of time on esterification reactions. However, other parameters were fixed at 9 wt% catalyst loading, a 1:16 oleic acid:methanol molar ratio, and 85 °C temperature. The highest yield of FAMEs, 99.01 ± 0.3% was found at a reaction time of 60 min (Fig. 7d). There was hardly any refinement in biodiesel yield after increasing the reaction time from 60 to 100 min. This phenomenon is described by the reversible esterification reaction and can be initiated by allowing the reaction duration to exceed its optimum value. As a consequence, the formed biodiesel was subsequently hydrolyzed^54^. Prolonged reaction times have been shown to reduce surface area through a decrease in active sites^54^. Therefore, the optimum reaction condition was fixed at 9 wt% catalyst loading, a 1:16 oleic acid:methanol molar ratio, 85 °C temperature, and 60 min reaction time for the microwave-assisted biodiesel production using WNS-SO_3_H heterogeneous acid catalyst.

### Comparative study of conventional and microwave-assisted biodiesel synthesis

**Figure 8:**
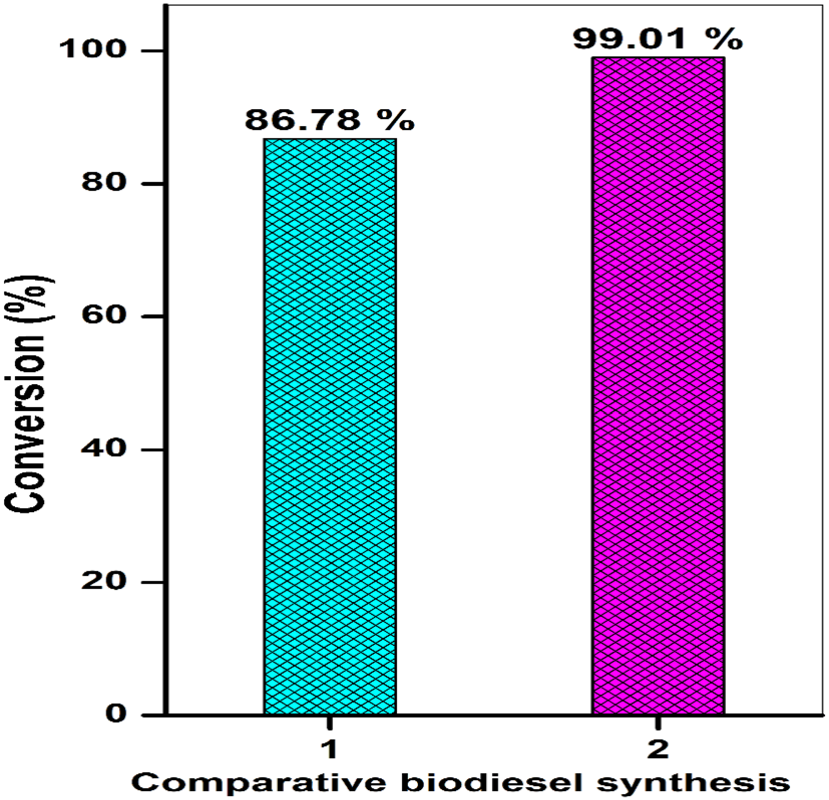
Comparative study of biodiesel synthesis, following conventional and microwave-assisted methods.

The catalytic performance of the WNS-SO_3_H catalyst was assessed by both conventional and microwave-assisted methods. The conventional method was used to perform the esterification reaction using the optimum conditions (9 wt% catalyst loading, a 1:16 oleic acid:methanol molar ratio, 85 °C temperature, and 60 min reaction time). For the conventional biodiesel synthesis, 9 wt% WNS-SO_3_H was taken in an ace pressure tube in 15 mL with a 1:16 molar ratio of oleic acid to methanol. The pressure tube was placed in a glycerol bath on a hot plate magnetic stirrer with the help of a holding stand. The conversion efficiency (%) obtained by conventional and microwave-assisted methods was 86.78 ± 0.6% and 99.01 ± 0.3%, respectively (Fig. 8). These findings are similar to the reported literature^26^. Furthermore, a comparison of the energy requirements of both the mechanically stirred reactor and the microwave-irradiated reactor was established. In our investigation, the power used by the microwave system during a 60 min reaction was 50 W, whereas, in a conventional mechanical stirred system, the same esterification reaction consumes 140 W of electricity over 60 min. As a result, the total energy used for the microwave-assisted esterification reaction was 90 W lower than the energy used for the mechanically stirred system, which meant that the consumption of energy in the microwave system would be even less. Furthermore, in comparison to previous studies, the energy and time consumption for the esterification of oleic acid with methanol in the presence of a WNS-SO_3_H catalyst is lower, resulting in higher conversion efficiency^55,56^.

### Comparison with previously reported –SO_3_H functionalized heterogeneous catalysts

Heterogenous acid catalysts show excellent catalytic activity for biodiesel production by the esterification process. Moreover, SFHCs are gaining much interest due to their excellent performance. Since most of the reported SFHCs were performed under conventional mechanically stirred esterification reactions. However, in this study, a microwave-assisted esterification process was carried out to achieve the highest yield of biodiesel (99.01 ± 0.3%). Table 2 represents the comparative study of the as-prepared WNS-SO_3_H catalyst, including specific parameters such as oleic acid:methanol molar ratio, reaction time, and reaction method. These significant parameters affect biodiesel production in terms of cost values. WNS-SO_3_H catalyst gives the highest biodiesel yield using a very low oleic acid:methanol molar ratio (1:16) and a very short reaction time compared to previously reported works^24,43^. The reaction time is very low compared to other studies employing microwave-assisted biodiesel production methods^55^.

**Table 2:** Comparison of WNS-SO_3_H catalyst with previously reported SFHCs.

SI. No.	Catalysts	Reaction conditions (Feedstock: methanol, time, temperature, and catalyst dosage)	Reaction method/reaction process	Biodiesel yield (%)	Refs.
1	SANH18	1:25, 60 min, 80 °C temperature, and 9 wt% catalyst	Microwave-assisted/esterification	96.40	^24^
2	SCPLB	1:20, 180 min, 70 °C temperature, and 5 wt% catalyst	Conventional/esterification	96.00	^26^
3	ASHC-SO_3_H	1:15, 180 min, 70 °C temperature, and 10 wt% catalyst	Conventional/esterification	96.04	^28^
4	C-SO_3_H	1:16, 240 min, 95 °C temperature, and 17 wt% catalyst	Conventional/transesterification	99.9	^57^
5	HZSM-5	1:45, 240 min, 100 °C temperature, and 10 wt% catalyst	Conventional/esterification	83.00	^58^
6	C450(120)-S90(120)	1:12, 180 min, 65 °C temperature, and 10 wt% catalyst	Microwave-assisted/esterification	98.1	^55^
7	OPT	1:18, 300 min, 65 °C temperature, and 9 wt% catalyst	Conventional/esterification	88.8	^59^
8	Biochar	1:30, 180 min, 315 °C temperature, and 5 wt% catalyst	Conventional/esterification	48.00	^60^
9	SAC-SCB	1:20, 360 min, 65 °C temperature, and 10 wt% catalyst	Conventional/esterification	85.1	^61^
10	WNS-SO_3_H	1:16, 60 min, 85 °C temperature, and 9 wt% catalyst	Microwave-assisted/esterification	99.01 ± 0.3%	This study

### Biodiesel characterization

**Figure 9:**
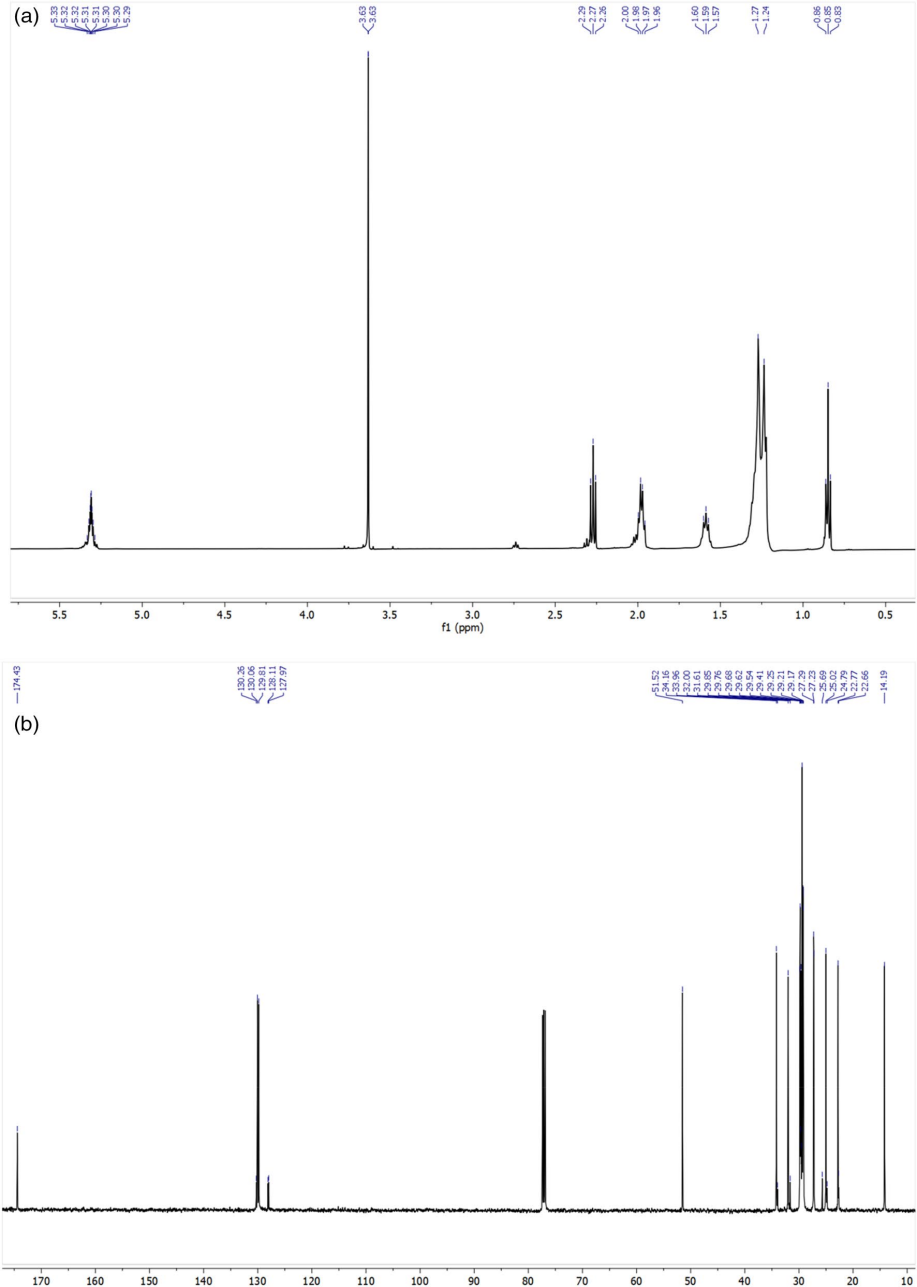
NMR spectrum of biodiesel obtained from the esterification of oleic acid with methanol catalyzed by WNS-SO_3_H using optimum reaction condition (**a**) ^1^H NMR spectrum (**b**) ^13^C NMR spectrum.

Microwave-assisted esterification reaction of oleic acid using methanol was performed in the presence of a WNS-SO_3_H catalyst to produce biodiesel. The final product of the reaction (biodiesel) was obtained employing optimum reaction parameters (9 wt% catalyst loading, a 1:16 oleic acid:methanol molar ratio, 85 °C temperature, and 60 min reaction time). Biodiesel was characterized after employing the purification process using a rotary evaporator. NMR and GC analysis was carried out for the biodiesel characterization, as described in “Characterization of biodiesel"” section. ^1^H and ^13^C nuclear magnetic resonance spectroscopy was carried out in a Jeol instrument having model number ECZ500R/S1 having a frequency ranging from 10 to 535 MHz. The biodiesel sample was dissolved in CDCl_3_ to perform the NMR analysis. Figure 9a and b portrayed the ^1^H NMR and ^13^C spectrum of biodiesel obtained by WNS-SO_3_H catalyzed esterification of oleic acid. The signal at 0.87 ppm is for the aliphatic –CH_3_ group present at the end of the chain. The signals at 1.27 and 1.6 ppm are related to aliphatic –CH_2_ groups away from double bonds and ester groups, while the signal at 2 ppm is for –CH_2_ groups adjacent to double bonds and 2.27 ppm represents the –CH_2_ groups (deshielded protons) adjacent to the carbonyl group of the ester. An intense peak at 3.6 ppm (Fig. 9a) reveals the presence of –OCH_3_ protons which confirms the product formation (biodiesel). Further, the signal at 5.31 ppm reveals the presence of an unsaturated system (–CH=CH–CH=CH–)^40,62^.

Figure 9b represents the ^13^C NMR spectrum of synthesized biodiesel in which a signal at 14.1 ppm corresponds to the –CH_3_ region. The signal in between 22.6 and 34.1 ppm revealing the –(CH_2_)n– region of biodiesel molecules. An intense signal at 51.5 ppm confirms the presence of the –COO–CH_3_ region. The signal at 127.9–130.2 ppm shows the presence of an unsaturated carbon region while the signal at 174.4 ppm for the carbonyl region.

**Figure 10:**
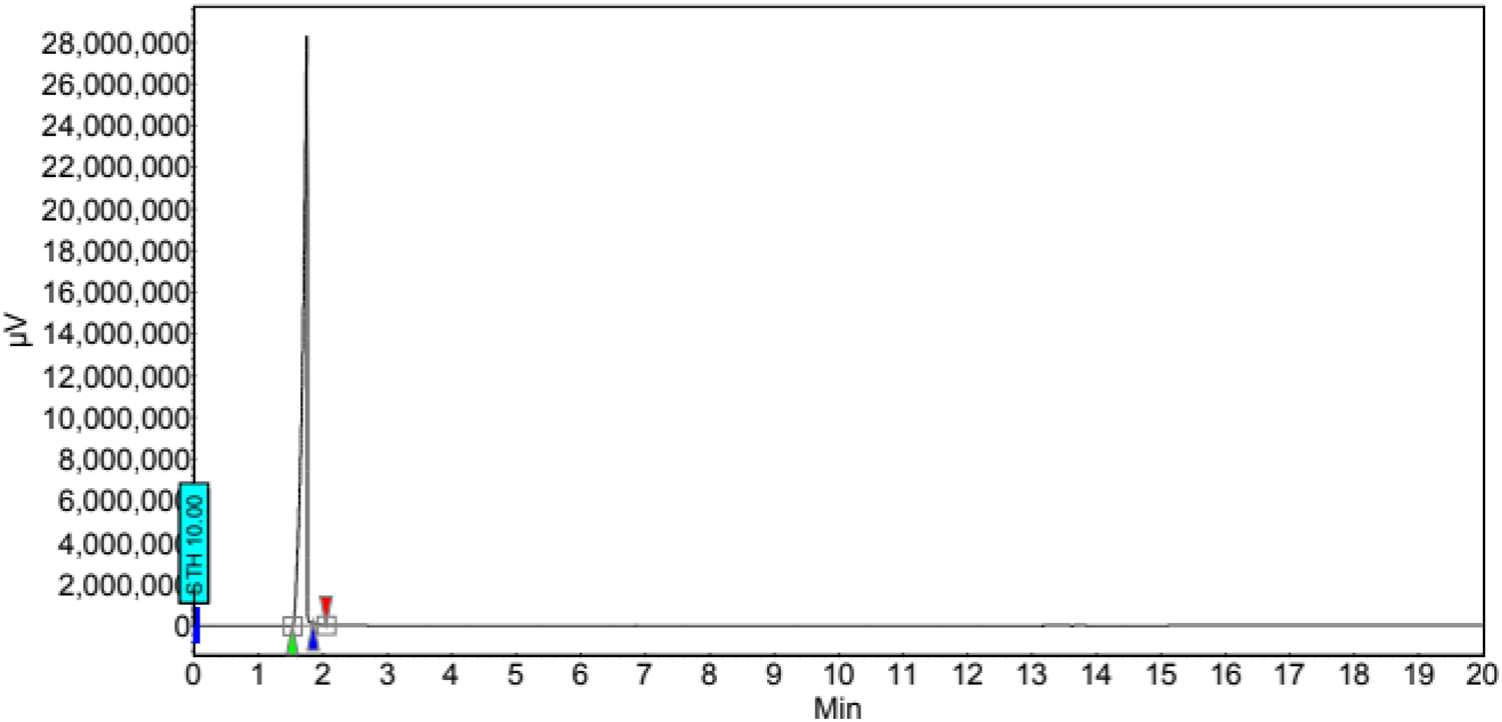
GC analysis spectrum of biodiesel synthesized using WNS-SO_3_H catalyst at optimum reaction conditions.

Figure 10 displayed the data obtained by GC analysis that confirms the biodiesel yield. The range of oven temperature during the GC analysis was 300–400 °C and pure methyl oleate was used as a standard.

### Chemical kinetics of esterification reaction

**Figure 11:**
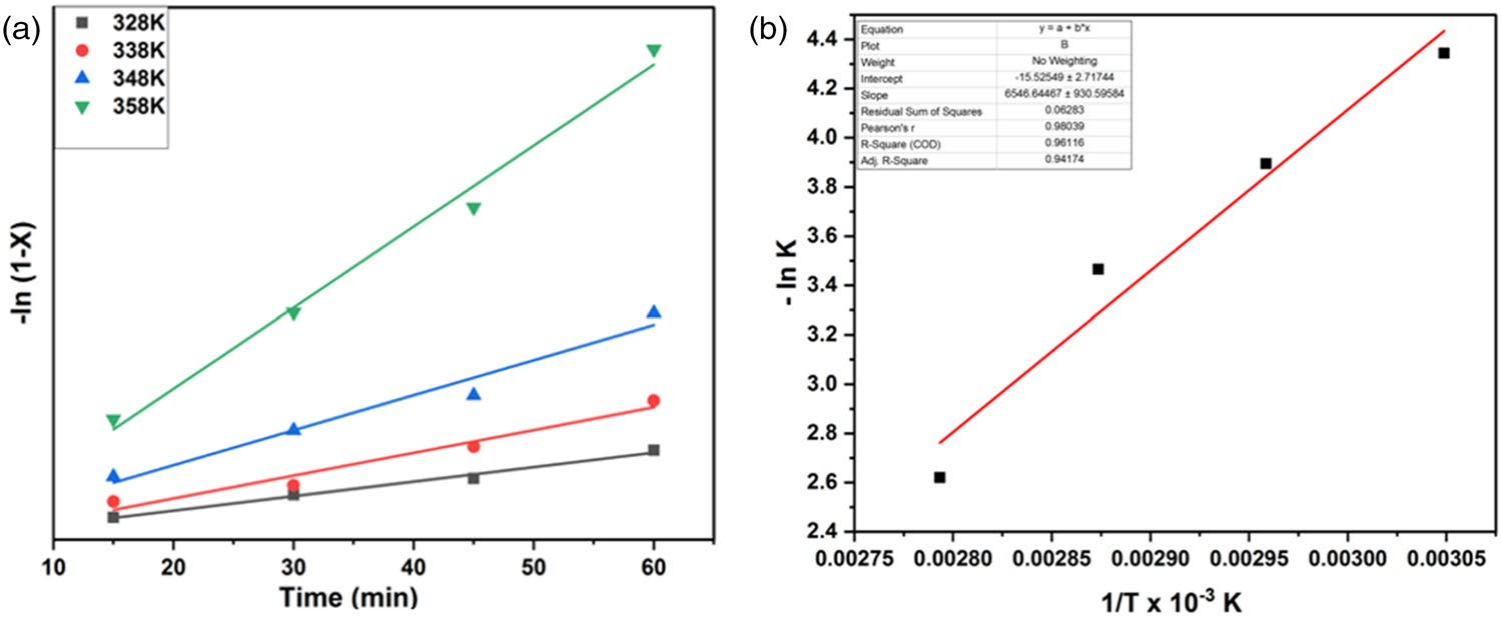
Kinetics study of esterification reaction using WNS-SO_3_H catalyst, (**a**) graphical plot of − ln(1 − X) versus t (X = Conversion of oleic acid) at different temperatures for the esterification of oleic acid with methanol, (**b**) the corresponding Arrhenius plot of − ln k versus 1/T.

The esterification of oleic acid is carried out by a WNS-SO_3_H catalyst in a homogeneous system where the overall reaction rate is controlled by the chemical reaction. By calculating the slope of − ln(1 − x) versus response time, we have calculated the perceptible rate constant (k). The resulting straight line is shown in Fig. 11a, and its R^2^ value of 0.96–0.98 confirms the feasibility of the pseudo-first-order reaction^34^. The activation energy was calculated by fitting the rate constant in the Arrhenius equation i.e., the value of lnk versus 1/T gives the activation energy and its slope value gives the pre-exponential factor (E_a_/R). The results from Fig. 11b show that the activation energy is 54.428 kJ mol^−1^ and the value of the pre-exponential factor is 5.4 × 10^–6^ min^−1^. The activation energies varied between 24.7 and 84.1 kJ mol^−1^ are appropriate for esterification reactions as mentioned in the literature^63,64^.

### Reusability of WNS-SO_3_H

**Figure 12:**
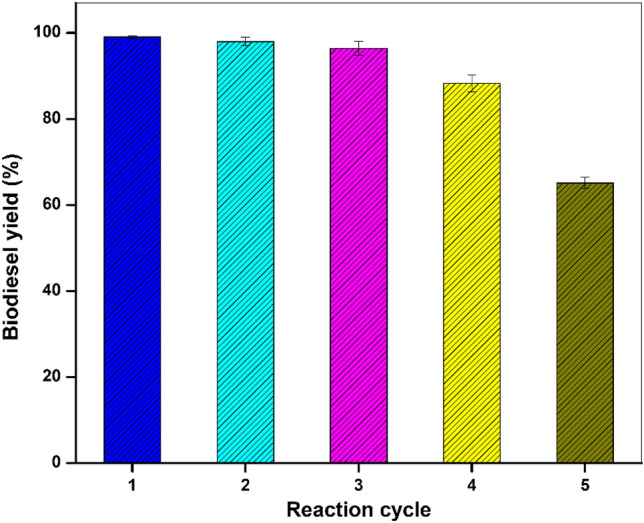
Reusability performance of WNS-SO_3_H catalyst.

Typically, the reusability of the WNS-SO_3_H catalyst has a significant impact on manufacturing costs independent of its catalytic capabilities. To assess the reusability of WNS-SO_3_H, the same catalyst was utilized five times in optimum reaction conditions. After every cycle, the catalyst was retrieved and rinsed with methanol 2–3 times and oven dried at 80 °C for 5 h before being reused again. A 99.01 ± 0.3% conversion was achieved in the first effective cycle of the esterification reaction using optimum conditions (9 wt% catalyst loading, a 1:16 oleic acid:methanol molar ratio, 85 °C temperature, and 60 min reaction time). For the next consecutive reaction cycles, the conversion obtained was 97.98%, 96.38%, and 88.24% for the 2nd, 3rd, and 4th cycles, respectively. Further, for the 5th cycle, conversion dropped significantly, and a 65.11% conversion yield was obtained. Figure 12 represents the results of catalyst reusability testing. After five reaction cycles, the catalyst became inactive as a result of the deactivation of the active sites (–SO_3_H groups) on the WNS-SO_3_H being deactivated and resulting in decreased total acid density^26^. In this work, the author hypothesizes that the primary deactivation process was the leaching of –SO_3_H groups; several other studies also showed similar behavior. However, the efficiency of the catalyst in the fifth cycle is equivalent to that reported in the literature^25,65^. Araujo et al.^25^ reported a significant decrease in the catalytic efficiency after 3 consecutive reaction cycles.

**Figure 13:**
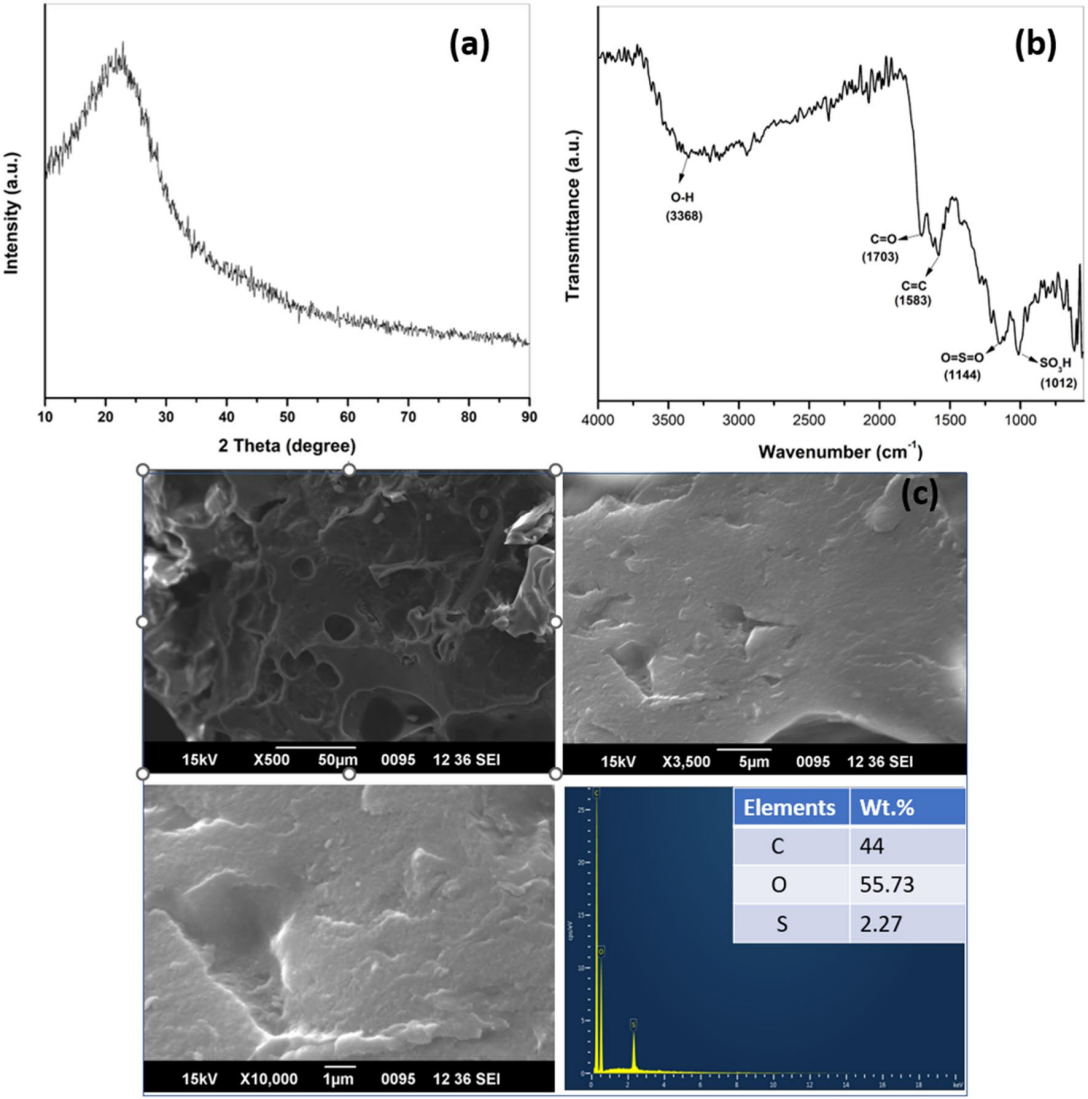
XRD pattern of RWNS (**a**), FTIR spectra of RWNS (**b**), SEM images of RWNS-SO_3_H at different magnifications along with EDS spectra show the elemental composition of RWNS-SO_3_H and reveal the decrease in sulfur content from the fresh catalyst (**c**).

After five reaction cycles the reused WNS-SO_3_H (RWNS) catalyst was characterized with XRD, FTIR, and SEM–EDS analysis to check out the structural, functional, and morphological changes. Figure 13a depicts the XRD pattern of RWNS and confirms that no structural changes occurred after the five consecutive reaction runs^11^. The FTIR spectra of RWNS (Fig. 13b) reveal the presence of all the functional groups –SO_3_H, O=S=O, C=O, C=C, and OH. However, the peaks related to these functional groups were slightly shifted from the peaks of fresh catalysts^24^. A decrease in the sulfur content from 4.76 to 2.27 wt% was observed by EDS analysis. Furthermore, the morphology of the catalyst was also changed and a pores structure can be seen in Fig. 13c. The removal of –SO_3_H sites promote the pores structure of the catalyst and favor the catalyst deactivation. Meanwhile, the total acid density of the WNS-SO_3_H catalyst decreased from 2.06 to 1.32 mmol/g after the fifth reaction cycle.

## Conclusion

The WNS-SO_3_H catalyst was prepared by low-temperature hydrothermal carbonization using lignin-rich biomass (walnut shell powder). The as-prepared catalyst provides the highest conversion yield (99.01 ± 0.3%) of biodiesel through microwave-assisted esterification reaction under the optimal reaction conditions. The obtained results show that the utilization of waste walnut shells solves environmental problems and effectively deals with sustainable energy. The WNS-SO_3_H catalyst can be employed for sustainable, eco-friendly, and cheap-cost production of biodiesel followed by the esterification of oleic acid. Moreover, the high amount of walnut shell waste and better reusability of the catalyst makes the catalyst appropriate for large-scale production.

## Data Availability

All data generated or analyzed during this study are included in this published article.
